# Spetzler-martin grade IV cerebral arteriovenous malformations in adult patients: a propensity-score matched analysis of resection and stereotactic radiosurgery

**DOI:** 10.1007/s10143-025-03465-6

**Published:** 2025-03-31

**Authors:** Salem M. Tos, Mahmoud Osama, Georgios Mantziaris, Bardia Hajikarimloo, Nimer Adeeb, Sandeep Kandregula, Hamza Adel Salim, Basel Musmar, Christopher S. Ogilvy, Douglas Kondziolka, Adam A. Dmytriw, Kareem El Naamani, Ahmed Abdelsalam, Deepak Kumbhare, Sanjeev Gummadi, Cagdas Ataoglu, Muhammed Amir Essibayi, Ufuk Erginoglu, Abdullah Keles, Sandeep Muram, Daniel Sconzo, Howard Riina, Arwin Rezai, Johannes Pöppe, Rajeev D. Sen, Louis J. Kim, Omar Alwakaa, Christoph J. Griessenauer, Pascal Jabbour, Stavropoula I. Tjoumakaris, Jan-Karl Burkhardt, Robert M. Starke, Mustafa K. Baskaya, Laligam N. Sekhar, Michael R. Levitt, David J. Altschul, Neil Haranhalli, Malia McAvoy, Abdallah Abushehab, Assala Aslan, Christian Swaid, Adib Abla, Christopher Stapleton, Matthew Koch, Visish M. Srinivasan, Peng R. Chen, Spiros Blackburn, Omar Choudhri, Bryan Pukenas, Darren Orbach, Edward Smith, Markus Möhlenbruch, Ali Alaraj, Ali Aziz-Sultan, Aman B. Patel, Amey Savardekar, Hugo H. Cuellar, Kathleen Dlouhy, Tarek El Ahmadieh, Michael Lawton, Adnan Siddiqui, Jacques Morcos, Bharat Guthikonda, Jason Sheehan

**Affiliations:** 1 Department of Neurosurgery, University of Virginia, Charlottesville, VA USA; 2 Department of Neurosurgery, Louisiana State University Health Science Center, Shreveport, LA USA; 3 Department of Neurosurgery, UT Health Sciences Center at Houston, McGovern Medical School, Houston, TX USA; 4 Department of Neurosurgery, University of Pennsylvania, Philadelphia, PA USA; 5 Department of Radiology, Louisiana State University, Shreveport, LA USA; 6 Department of Neurosurgery, Thomas Jefferson University Hospital, Philadelphia, PA USA; 7 Division of Neurosurgery, Beth Israel Deaconess Medical Center, Harvard Medical School, Boston, MA USA; 8 Department of Neurosurgery, New York University Grossman School of Medicine, Manhattan, NY USA; 9 Neuroendovascular Program, Massachusetts General Hospital, Harvard Medical School, Boston, MA USA; 10 Department of Neurosurgery, University of Miami, Miller School of Medicine, Miami, FL USA; 11 Department of Neurosurgery, University of Wisconsin School of Medicine, Madison, WI USA; 12 Montefiore Einstein Cerebrovascular Research Lab, Department of Neurological Surgery, Montefiore Medical Center, Albert Einstein College of Medicine, NY, USA; 13 Department of Neurosurgery, Christian Doppler Klinik, Paracelsus Medical University, Salzburg, Austria; 14 Department of Neurosurgery, University of Washington, Seattle, WA USA; 15 Department of Plastic Surgery, Mayo Clinic Hospital, Rochester, MN USA; 16 Department of Neurosurgery, University of Florida, Gainesville, FL USA; 17 Neurointerventional Radiology, Boston Children’s Hospital, Harvard Medical School, Boston, MA USA; 18 Department of Neurosurgery, Boston Children’s Hospital, Harvard Medical School, Boston, MA USA; 19 Interventional Neuroradiology, Department of Neuroradiology, Heidelberg University Hospital, Heidelberg, Germany; 20 Department of Neurosurgery, University of Illinois in Chicago, Chicago, IL USA; 21 Department of Neurosurgery, Brigham and Women’s Hospital, Harvard Medical School, Boston, MA USA; 22 Department of Neurosurgery, University of Iowa, Iowa City, IA USA; 23 Department of Neurosurgery, Loma Linda University, Redlands, CA USA; 24 Department of Neurosurgery, Barrow Neurological Institute, Phoenix, AZ USA; 25 Department of Neurosurgery, State University of New York at Buffalo, Buffalo, NY USA; 26 Department of Neurological Surgery, University of Virginia, Box 800212, Charlottesville, VA 22908 USA

**Keywords:** Cerebral arteriovenous malformations, Spetzler-martin grade IV, Resection, Stereotactic radiosurgery, AVM obliteration, Complication rates and functional outcomes

## Abstract

Spetzler-Martin Grade IV arteriovenous malformations (AVMs) are challenging due to high risks associated with both treatment and natural progression. This study compares the outcomes of microsurgical resection and stereotactic radiosurgery (SRS) in high-grade AVMs, analyzing obliteration rates, complications, and functional outcomes. A retrospective cohort of 96 patients treated with either microsurgical resection (33 patients) or SRS (63 patients) was analyzed. Propensity-score matching was employed to account for baseline variables such as AVM size (cm), preoperative embolization and rupture status. Primary endpoints included AVM obliteration, complication rates, and modified Rankin Scale (mRS) scores. After matching, 31 patients per group were analyzed. Microsurgical resection achieved significantly higher obliteration rates (87.1%) compared to SRS (32.3%, *p* < 0.001). In the matched SRS cohort (*n* = 31), the actuarial obliteration rates were 11% (95% CI: 0-22%) at 1 year, 17% (95% CI: 0-31%) at 3 years, and 43% (95% CI: 13-63%) at 5 years post-treatment. Complication rates were similar (32.3% resection, 38.7% SRS, *p* = 0.6). Functional outcomes in terms of improvement in modified Rankin Scale (mRS) scores were observed in 50.0% of microsurgery patients and 41.4% of SRS patients. However, the absolute number of patients improving was similar (13 vs. 12), and the microsurgery group had more cases of worsening mRS scores compared to the SRS group (4 vs. 2). The difference was not statistically significant (*p* = 0.4). Microsurgical resection offers superior obliteration rates for high-grade AVMs with comparable complication risks to SRS. SRS remains a valuable alternative for select patients, particularly those ineligible for resection. Future research should focus on optimizing multimodal treatment approaches.

**Clinical trial number** Not applicable.

**Graphical Abstract:**

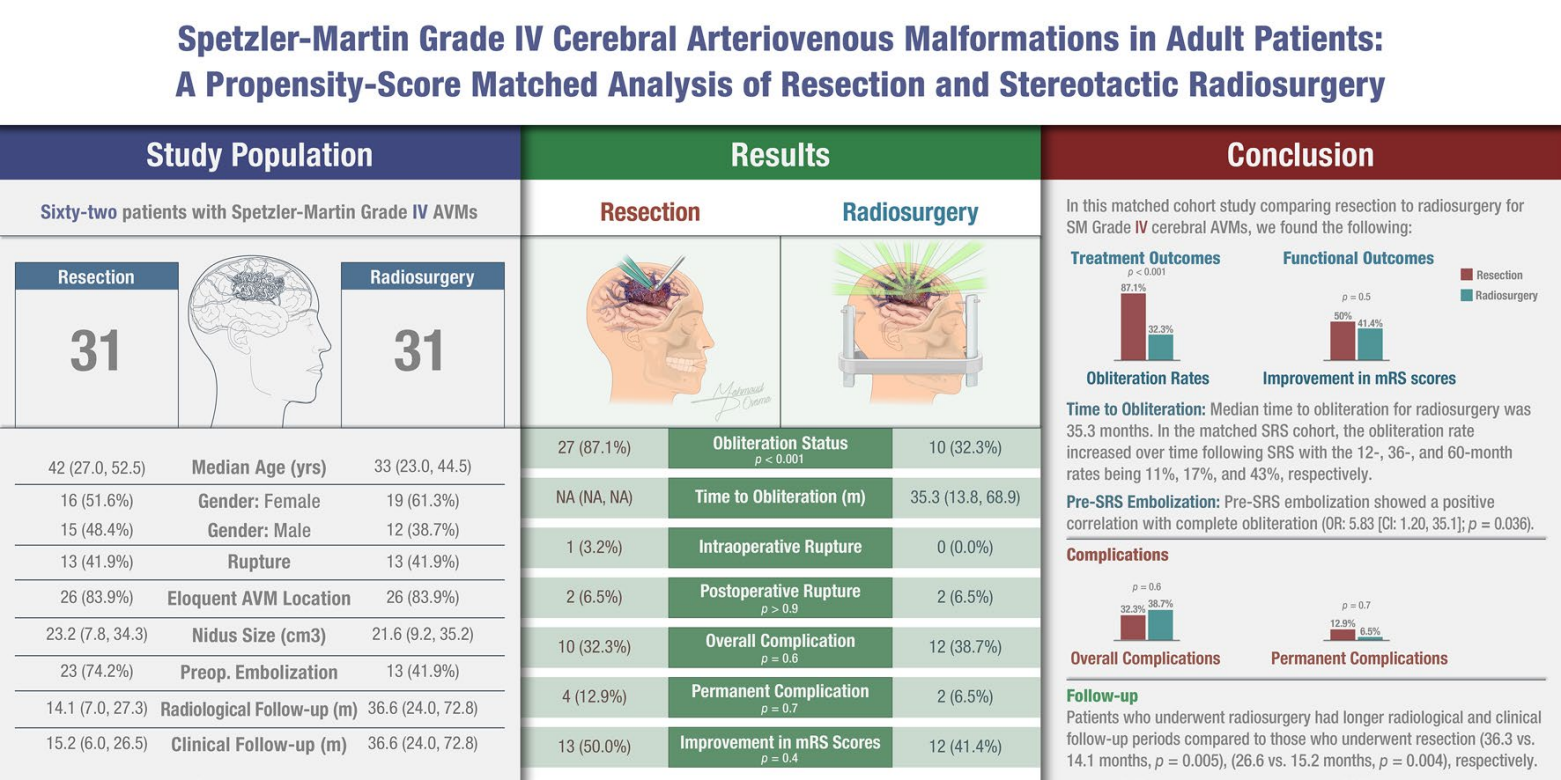

## Introduction

Cerebral arteriovenous malformations (AVMs) are vascular lesions with disrupted, direct connection between arteries and veins, bypassing the capillary network. These lesions pose significant risks, particularly due to their potential for rupture, causing intracranial haemorrhage and devastating neurological damage [1, 2]. The incidence of AVMs reported between 1.12 and 1.34 per 100,000 person-years, a number consistent across all population studies [3, 4]. The risk of rupture for an AVM is 2–4% annually, although that is particularly so for large or complex lesions found in the eloquent areas of the brain [4–6]. Consequently, the management of AVMs is highly individualized and challenging, with a careful balance between intervention risks and the natural course of the disease [7].

**Fig. 1:**
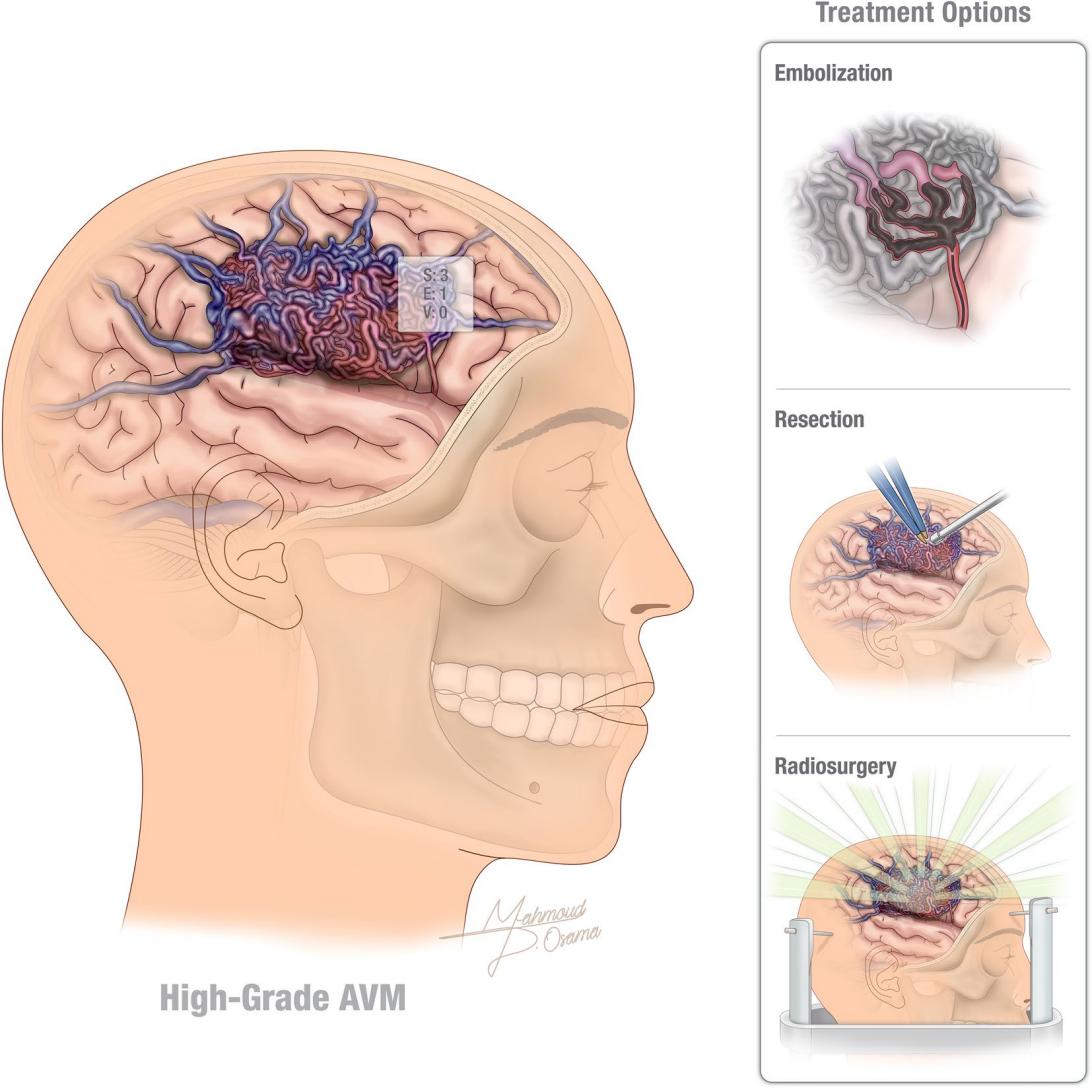
High-grade brain arteriovenous malformation (AVM) with treatment options: embolization, resection, and radiosurgery

Several treatment options are available, including conservative management (observation), microsurgical resection, stereotactic radiosurgery (SRS), and endovascular embolization, used alone or in combination [8, 9]. Treatment choice is based on age, AVM size, location, angioarchitecture, and the degree of rupture or unrupture [10]. Historically, microsurgical resection has been regarded as the gold standard for achieving immediate AVM obliteration, especially in low-grade lesions. However, high-grade AVMs, specifically Spetzler-Martin (SM) Grades IV, present substantial treatment challenges due to their size, location, and involvement of critical brain regions (Fig. 1). [11, 12]

The role of SRS and/or resection for SM IV AVM’s remains incompletely defined. Resection, though often associated with higher obliteration rates, carries significant morbidity risks, especially in larger, more complex lesions. On the other hand, while SRS offers a less invasive alternative, its efficacy in obliterating high-grade AVMs is often lower, with obliteration rates ranging between 15% and 46% for SM Grades IV and V [11, 13–15]. In the past three decades, SRS has gained popularity as an alternative, particularly for patients with AVMs deemed unsuitable for surgery [16–18]. SRS offers a non-invasive approach, delivering precise radiation to the AVM nidus and promoting gradual obliteration of the lesion over time [11, 19]. This delayed effect, however, carries its own set of risks, including a latency period during which patients remain at risk of hemorrhage [20]. Additionally, complications such as radiation-induced changes and post-SRS hemorrhages during the latency period further complicate the management of these patients [15, 21].

Given these complexities, there is a clear need for comprehensive studies that analyze outcomes of SRS and resection in SM Grade IV AVMs. This study aims to address this gap in the literature by providing a detailed comparison of SRS and resection, focusing on obliteration rates, complication profiles, and long-term outcomes.

## Methods

### Study design

This study’s data are a subset of the Multicenter International Study for Treatment of Brain AVMs (MISTA) database, focusing on adult patients that underwent resection or SRS for SM grade IV brain AVMs. MISTA was established in 2024 as a collaborative effort among 12 high-volume neurovascular centers across North America and Europe. The database was created through systematic retrospective collection of consecutive AVM cases treated between 2010 and 2024 via different treatments including resection, radiosurgery, embolization or combination, with standardized data entry protocols, for the diagnosis, treatment, and follow-up of AVM patients, to minimize selection bias. Each participating center contributed all brain AVM cases meeting predefined criteria, regardless of treatment outcomes. Regular data audits and standardized definitions ensure consistency across centers. The study included patients with SM Grade IV AVMs, with or without prior embolization. Exclusion criteria were incomplete records, SM Grade I-III and V AVMs. Institutional review boards approved the protocol, waiving informed consent due to the retrospective nature. Data were de-identified for confidentiality. The study followed STROBE guidelines for observational studies to ensure robust and reproducible findings.

### Data source and variables

The study collected comprehensive patient data encompassing demographics (age, gender, race), clinical presentation (initial symptoms at AVM diagnosis), and detailed AVM characteristics. These included rupture status, anatomical location, eloquence, nidus diameter and volume, SM grade, vascular features (arterial feeders, draining veins), and associated vascular anomalies (venous stenosis, aneurysms). Deep-seated AVMs are defined as those located in the subcortical lobes, corpus callosum, basal ganglia, thalamus, brainstem, insula, or deep cerebellum.[22–24] In the matched cohort analysis, deep located AVMs were equal in each cohort. Treatment timing was categorized as: within 24 h, 7 days, 7–14 days, and after 14 days from rupture. This thorough data collection enabled a detailed analysis of patient profiles and AVM features in relation to treatment outcomes.

### Surgical modality, follow-up, and outcomes

The management of AVMs in this study involved distinct treatment pathways for resection and SRS, with some patients having undergone prior embolization. Microsurgical resection was performed according to the operating neurosurgeon’s clinical judgment, with patients subsequently receiving specialized care within a neurological inpatient setting. Conversely, SRS involved recording the specific modality employed (Gamma Knife, CyberKnife, or linear accelerator) and the prescribed radiation dose. SRS treatments included both single-session and volume-staged approaches, depending on the characteristics of the AVM and the treating physician’s assessment. While general guidelines were followed to ensure consistency, there were inherent variations in specific practices, reflecting real-world clinical diversity. Diagnosis was standardized using imaging modalities such as MRI/MRA, DSA, CTA to confirm AVM characteristics and suitability for treatment. Treatment protocols, including resection and stereotactic radiosurgery, were guided by institutional expertise and patient-specific factors. Follow-up neuroimaging (MRI/MRA, DSA, CTA) and clinical assessments, were performed according to each center’s routine practices and patient-specific considerations, with variability in the timing and type of imaging. Patients treated with SRS were monitored during the latency period for potential complications, including hemorrhage, through regular clinical evaluations and imaging studies, including MRI or CT scans. While each institution adhered to general guidelines for post-SRS care, the frequency and specifics of follow-up protocols were not fully standardized across all centers. This variability reflects real-world clinical practice and highlights the external validity of the study but also represents a limitation in achieving complete consistency across institutions. Radiation-induced changes were identified on follow-up neuroimaging as perinidal hyperintensities or cyst formation and were classified as permanent based on the persistence of associated neurological symptoms for more than 6 months.

### Statistical analysis

The study employed R version 4.3.2 (2024) for statistical analysis. Descriptive statistics were computed for all variables, with continuous data presented as medians and interquartile ranges, and categorical data as frequencies and percentages. The resection and SRS groups were compared using Wilcoxon rank-sum tests for continuous variables and Chi-squared or Fisher’s exact tests for categorical variables. To assess obliteration rates, time to obliteration, surgical complications, and neurological outcomes, propensity score matching was utilized. This technique created 1:1 matched cohorts based on nidus size (cm), preoperative embolization and rupture status. The MatchIt” package in R performed optimal matching for propensity score variables and exact matching for rupture status and location. To assess the balance of covariates between treatment groups, standardized mean differences (SMD) were calculated for each variable before and after matching. A threshold of SMD < 0.1 was used to define good balance. After matching, the covariates nidus size (cm) and rupture status achieved good balance, with SMD values below 0.1. However, the covariate preoperative embolization achieved only moderate balance after matching, with an SMD between 0.1 and 0.2. Logistic regression analyzed complete obliteration and symptomatic complications, with statistical significance defined as p < 0.05.

## Results

### Patient, AVM, and procedural characteristics: unmatched cohorts

The study included 96 patients: 33 in the resection group and 63 in the radiosurgery group. (Table 1) Significant differences were observed between the groups. The resection cohort was older (median 42.0 vs. 33.0 years, *p* = 0.022) and more likely to undergo treatment within 24 h of rupture (54.5% vs. 15.8%, *p* = 0.030). Resection patients had a higher prevalence of parietal AVMs (39.4% vs. 17.5%, *p* = 0.018), more compacted AVMs (84.4% vs. 55.7%, *p* = 0.006), and higher rates of preoperative embolization (75.8% vs. 28.6%, *p* < 0.001). However, clinical presentation, presentation mRS, gender distribution, AVM rupture rates, nidus size, and SM grades were similar between groups. Patients in the radiosurgery group had significantly longer follow-up periods compared to the resection group, both radiologically (29.4 vs. 13.1 months, *p* = 0.017) and clinically (27.5 vs. 13.1 months, *p* = 0.028).

**Table 1:** Comparison of the patient, AVM, and procedural characteristics of the resection and stereotactic radiosurgery cohorts

	Original Data				Matched Data			
Characteristic	**Overall***N* = 96^*1*^	**Resection***N* = 33^*1*^	**Radiosurgery***N* = 63^*1*^	**p-value** ^*2*^	**Overall***N* = 62^*1*^	**Resection***N* = 31^*1*^	**Radiosurgery***N* = 31^*1*^	**p-value** ^*2*^
**Age, years**	33.0 (23.8,48.0)	42.0 (27.0,52.0)	29.0 (21.5,42.0)	0.022	35.5 (24.3,50.8)	42.0 (27.0,52.5)	33.0 (23.0,44.5)	0.11
**Gender**				> 0.9				0.4
Female	49 (51.0%)	17 (51.5%)	32 (50.8%)		35 (56.5%)	16 (51.6%)	19 (61.3%)	
Male	47 (49.0%)	16 (48.5%)	31 (49.2%)		27 (43.5%)	15 (48.4%)	12 (38.7%)	
**Race**				0.5				> 0.9
Asian	1 (1.6%)	0 (0.0%)	1 (2.9%)					
Black	5 (7.9%)	3 (10.7%)	2 (5.7%)		5 (12.2%)	3 (11.5%)	2 (13.3%)	
Hispanic	19 (30.2%)	10 (35.7%)	9 (25.7%)		14 (34.1%)	9 (34.6%)	5 (33.3%)	
Native American	1 (1.6%)	1 (3.6%)	0 (0.0%)		1 (2.4%)	1 (3.8%)	0 (0.0%)	
White	35 (55.6%)	14 (50.0%)	21 (60.0%)		21 (51.2%)	13 (50.0%)	8 (53.3%)	
Others	35	5	30		21	5	16	
**Clinical Presentation at diagnosis**				0.059				0.4
None	11 (11.5%)	6 (18.2%)	5 (7.9%)		7 (11.3%)	5 (16.1%)	2 (6.5%)	
Headache	36 (37.5%)	9 (27.3%)	27 (42.9%)		22 (35.5%)	9 (29.0%)	13 (41.9%)	
Seizure	25 (26.0%)	7 (21.2%)	18 (28.6%)		14 (22.6%)	6 (19.4%)	8 (25.8%)	
Cerebellar	2 (2.1%)	2 (6.1%)	0 (0.0%)		2 (3.2%)	2 (6.5%)	0 (0.0%)	
Hemorrhage	8 (8.3%)	5 (15.2%)	3 (4.8%)		7 (11.3%)	5 (16.1%)	2 (6.5%)	
Motor deficits	9 (9.4%)	2 (6.1%)	7 (11.1%)		5 (8.1%)	2 (6.5%)	3 (9.7%)	
Speech deficits	1 (1.0%)	1 (3.0%)	0 (0.0%)		1 (1.6%)	1 (3.2%)	0 (0.0%)	
Visual Disturbance	2 (2.1%)	0 (0.0%)	2 (3.2%)		2 (3.2%)	0 (0.0%)	2 (6.5%)	
Confusion	2 (2.1%)	1 (3.0%)	1 (1.6%)		2 (3.2%)	1 (3.2%)	1 (3.2%)	
**Presentation mRS**				0.6				0.8
0	37 (38.5%)	13 (39.4%)	24 (38.1%)		21 (33.9%)	11 (35.5%)	10 (32.3%)	
1	14 (14.6%)	4 (12.1%)	10 (15.9%)		8 (12.9%)	4 (12.9%)	4 (12.9%)	
2	17 (17.7%)	6 (18.2%)	11 (17.5%)		15 (24.2%)	6 (19.4%)	9 (29.0%)	
3	12 (12.5%)	2 (6.1%)	10 (15.9%)		5 (8.1%)	2 (6.5%)	3 (9.7%)	
4	8 (8.3%)	4 (12.1%)	4 (6.3%)		5 (8.1%)	4 (12.9%)	1 (3.2%)	
5	8 (8.3%)	4 (12.1%)	4 (6.3%)		8 (12.9%)	4 (12.9%)	4 (12.9%)	
**History of hemorrhage prior to treatment (Ruptured AVM)**	39 (40.6%)	13 (39.4%)	26 (41.3%)	0.9	26 (41.9%)	13 (41.9%)	13 (41.9%)	> 0.9
**Time from rupture to treatment**				0.030				0.006
24 h	9/30 (30.0%)	6/11 (54.5%)	3/19 (15.8%)		8/22 (36.4%)	6/11 (54.5%)	2/11 (18.2%)	
<7d	2/30 (6.7%)	1/11 (9.1%)	1/19 (5.3%)		2/22 (9.1%)	1/11 (9.1%)	1/11 (9.1%)	
7d-14d	0/30 (0.0%)	0/11 (0.0%)	0/19 (0.0%)		0/22 (0.0%)	0/11 (0.0%)	0/11 (0.0%)	
>14d	19/30 (63.3%)	4/11 (36.4%)	15/19 (78.9%)		12/22 (54.5%)	4/11 (36.4%)	8/11 (72.7%)	
**Location**								
Frontal	35 (36.5%)	12 (36.4%)	23 (36.5%)	> 0.9	22 (35.5%)	11 (35.5%)	11 (35.5%)	> 0.9
Temporal	13 (13.5%)	2 (6.1%)	11 (17.5%)	0.2	10 (16.1%)	2 (6.5%)	8 (25.8%)	0.038
Parietal	24 (25.0%)	13 (39.4%)	11 (17.5%)	0.018	18 (29.0%)	13 (41.9%)	5 (16.1%)	0.025
Occipital	15 (15.6%)	7 (21.2%)	8 (12.7%)	0.3	10 (16.1%)	7 (22.6%)	3 (9.7%)	0.2
Cerebellar	2 (2.1%)	2 (6.1%)	0 (0.0%)	0.12	2 (3.2%)	2 (6.5%)	0 (0.0%)	0.5
Corpus Callosum	2 (2.1%)	0 (0.0%)	2 (3.2%)	0.5	1 (1.6%)	0 (0.0%)	1 (3.2%)	> 0.9
Insular	1 (1.0%)	0 (0.0%)	1 (1.6%)	> 0.9				
Thalamus	11 (11.5%)	1 (3.0%)	10 (15.9%)	0.091	5 (8.1%)	1 (3.2%)	4 (12.9%)	0.4
Basal Ganglia	5 (5.2%)	1 (3.0%)	4 (6.3%)	0.7	3 (4.8%)	1 (3.2%)	2 (6.5%)	> 0.9
Brainstem	2 (2.1%)	0 (0.0%)	2 (3.2%)	0.5	1 (1.6%)	0 (0.0%)	1 (3.2%)	> 0.9
**Eloquent Area**	56 (55.3%)	16 (48.5%)	37 (58.8%)	0.3	39 (62.9%)	16 (51.6%)	23 (74.2%)	0.8
**Deep^**	49 (51.0%)	15 (45.5%)	34 (54.0%)	0.4	28 (45.2%)	14 (45.2%)	14 (45.2%)	> 0.9
**Nidus diameter (cm)**	4.3 (3.7,5.7)	4.3 (3.6,6.2)	4.3 (3.7,5.5)	0.6	4.4 (3.6,6.2)	4.3 (3.6,6.2)	4.5 (3.8,6.0)	0.7
**Volume (cm** ^**3**^ **)**	22.6 (10.5,48.4)	23.2 (9.6,36.2)	22.5 (11.2,49.2)	0.5	21.7 (9.1,35.4)	23.2 (7.8,34.3)	21.6 (9.2,35.2)	> 0.9
Spetzler Martin grade IV subtypes*				0.3				0.13
S2E1V1	47 (49.0%)	13 (39.4%)	34 (54.0%)		33 (53.2%)	13 (41.9%)	20 (64.5%)	
S3E0V1	43 (44.8%)	17 (51.5%)	26 (41.3%)		23 (37.1%)	15 (48.4%)	8 (25.8%)	
S3E1V0	6 (6.3%)	3 (9.1%)	3 (4.8%)		6 (9.7%)	3 (9.7%)	3 (9.7%)	
**Modified Pollock-Flickinger AVM Score**				NA				NA
I	3 (4.8%)	—	3 (4.8%)		3 (9.7%)	—	3 (9.7%)	
II	5 (7.9%)	—	5 (7.9%)		3 (9.7%)	—	3 (9.7%)	
III	7 (11.1%)	—	7 (11.1%)		3 (9.7%)	—	3 (9.7%)	
IV	48 (76.2%)	—	48 (76.2%)		22 (70.9%)	—	22 (70.9%)	
**Compacted**	61 (65.6%)	27 (84.4%)	34 (55.7%)	0.006	37 (61.7%)	25 (83.3%)	12 (40.0%)	< 0.001
**Number of Feeders**				0.6				0.5
Multiple	93 (95.9%)	31 (93.9%)	62 (96.9%)		56 (96.6%)	27 (93.1%)	29 (100.0%)	
Single	4 (4.1%)	2 (6.1%)	2 (3.1%)		2 (3.4%)	2 (6.9%)	0 (0.0%)	
**Number of Draining Veins**				0.4				0.6
Multiple	52 (89.7%)	22 (84.6%)	30 (93.8%)		32 (86.5%)	20 (83.3%)	12 (92.3%)	
Single	6 (10.3%)	4 (15.4%)	2 (6.3%)		5 (13.5%)	4 (16.7%)	1 (7.7%)	
**Location of Draining Veins**				0.4				0.11
Both	33 (34.4%)	13 (39.4%)	20 (31.7%)		17 (27.4%)	12 (38.7%)	5 (16.1%)	
Deep	57 (59.4%)	17 (51.5%)	40 (63.5%)		39 (62.9%)	16 (51.6%)	23 (74.2%)	
Superficial	6 (6.3%)	3 (9.1%)	3 (4.8%)		6 (9.7%)	3 (9.7%)	3 (9.7%)	
**Venous Stenosis**	2 (3.3%)	0 (0.0%)	2 (5.9%)	0.5	1 (2.6%)	0 (0.0%)	1 (7.1%)	0.4
**Aneurysm**	31 (32.3%)	12 (36.4%)	19 (30.2%)	0.5	23 (37.1%)	12 (38.7%)	11 (35.5%)	0.8
**Preoperative Embolization**	43 (44.8%)	25 (75.8%)	18 (28.6%)	< 0.001	36 (58.1%)	23 (74.2%)	13 (41.9%)	0.010
**Embolization to surgery time, days**	30.0 (6.5,100.5)	19.0 (5.0,30.0)	117.0 (84.8,198.5)	< 0.001	30.0 (5.0,61.0)	12.0 (4.0,30.0)	105.0 (68.3,198.5)	< 0.001
**SRS Modality**				> 0.9				> 0.9
Cyberknife	11 (17.5%)	—	11 (17.5%)		6 (19.4%)	—	6 (19.4%)	
GKRS	45 (71.4%)	—	45 (71.4%)		20 (64.5%)	—	20 (64.5%)	
LINAC	7 (11.1%)	—	7 (11.1%)		5 (16.1%)	—	5 (16.1%)	
**Prescription Dose (Gy)**	17.0 (16.0,18.0)	—	17.0 (16.0,18.0)		17.5 (16.0,19.8)	—	17.5 (16.0,19.8)	
**Number of SRS stages**				> 0.9				> 0.9
1	38 (60.3%)	—	38 (60.3%)		20 (64.5%)	—	20 (64.5%)	
2	24 (38.1%)	—	24 (38.1%)		10 (32.3%)	—	10 (32.3%)	
3	1 (1.6%)	—	1 (1.6%)		1 (3.2%)	—	1 (3.2%)	
**Radiological follow-up, months**	24.0 (12.0,43.5)	13.1 (8.0,26.5)	29.4 (17.9,47.1)	0.017	25.4 (12.0,56.3)	14.1 (7.0,27.3)	36.6 (24.0,72.8)	0.005
**Clinical follow-up, months**	24.0 (11.6,43.5)	13.1 (6.0,26.0)	27.5 (16.5,47.1)	0.028	25.4 (11.8,60.0)	15.2 (6.0,26.5)	36.6 (24.0,72.8)	0.004

^*1*^Median (25%,75%); n (%)

^*2*^Wilcoxon rank sum test; Pearson’s Chi-squared test; Fisher’s exact test

* S: Size (S2: Medium 3–6 cm; S3: Large > 6 cm), E: Eloquence (E0: Non-eloquent; E1: Eloquent brain), V: Venous drainage (V0: Superficial only; V1: Deep)

^ Deep-seated AVMs are defined as those located in the subcortical lobes, corpus callosum, basal ganglia, thalamus, brainstem, insula, or deep cerebellum

### Patient, AVM, and procedural characteristics: matched cohorts

After matching, the cohort included 62 patients, with 31 in each treatment group. (Table 1) The matching process improved balance for many characteristics, but some differences persisted. Age disparity was reduced (42.0 vs. 33.0 years, *p* = 0.11), and gender (*p* = 0.4) and race distributions (*p* > 0.9) became well-balanced. Clinical presentation (*p* = 0.4) and AVM rupture rates (41.9% for both groups) were equalized. However, significant differences remained in rupture timing (*p* = 0.006), parietal AVM location (41.9% vs. 16.1%, *p* = 0.025), and use of preoperative embolization (74.2% vs. 41.9%, *p* = 0.01). The difference in compacted AVMs was still notable (83.3% resection vs. 40.0% radiosurgery, *p* < 0.001). Patients in the radiosurgery group had significantly longer follow-up periods compared to the resection group, both radiologically (36.6.4 vs. 14.1 months, *p* = 0.005) and clinically (36.6 vs. 15.2 months, *p* = 0.004).

The Modified Pollock-Flickinger AVM Score distribution showed that in our cohort, Class IV was predominant. Among all cases, 48 patients (76.2%) were classified as Class IV, while Class III included 7 patients (11.1%). Class II comprised 5 patients (7.9%), and Class I had the smallest representation with 3 patients (4.8%). In the matched cohort analysis, a similar distribution pattern was observed, with Class IV remaining the largest group at 22 patients (70.9%), while Classes I, II, and III each contained 3 patients (9.7%).

### Obliteration rate

**Fig. 2:**
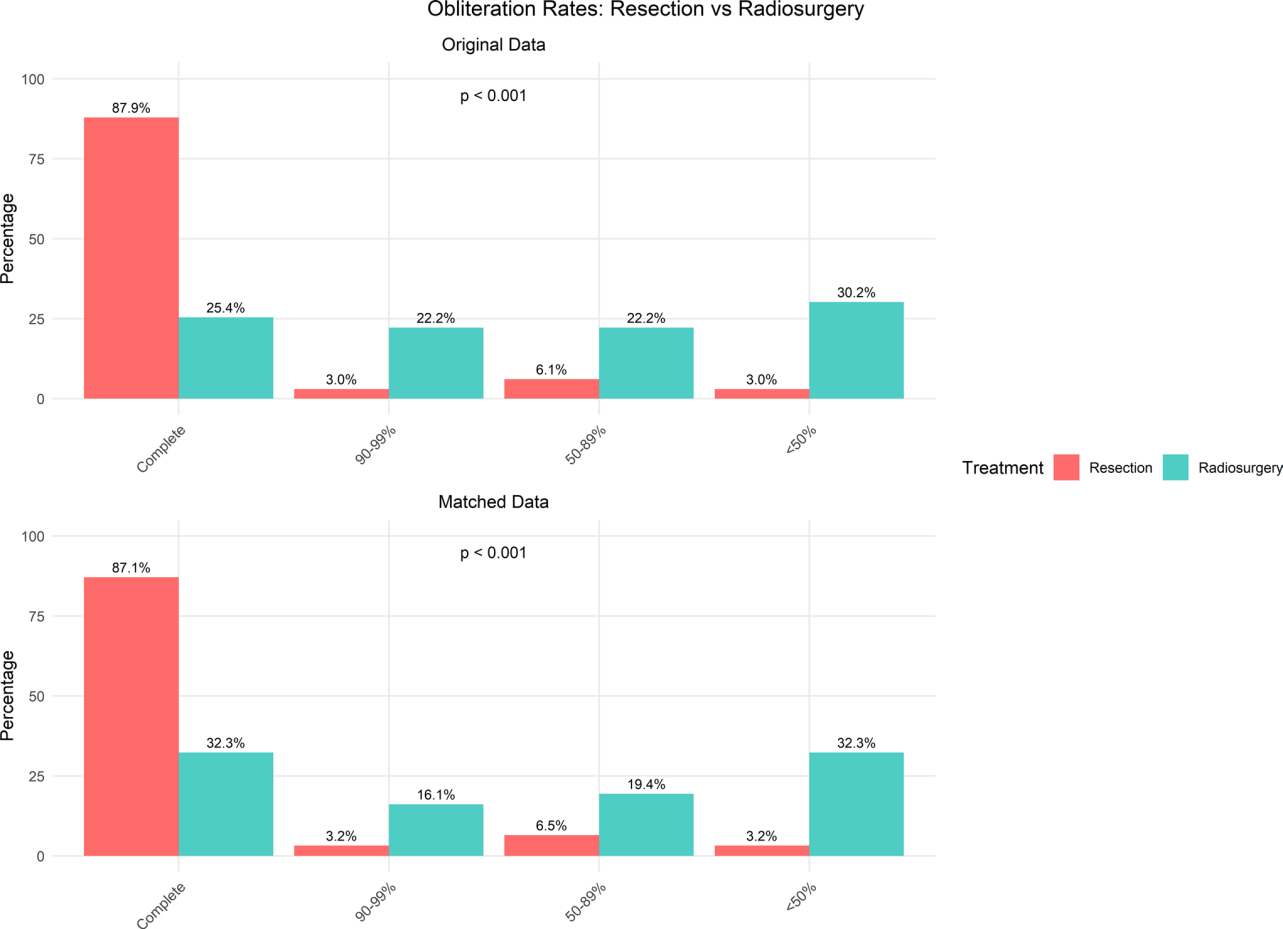
Comparison of obliteration status between resection and stereotactic radiosurgery (SRS) cohorts, presented for both original and matched data. Resection shows a higher complete obliteration rate (*p* < 0.01)

Before matching, resection achieved complete obliteration in 29 out of 33 patients (87.9%), while radiosurgery resulted in complete obliteration in only 16 out of 63 patients (25.4%), a statistically significant difference (*p* < 0.001). (Fig. 2; Table 2) After matching, this disparity persisted, with 27 out of 31 patients (87.1%) achieving obliteration after resection compared to 10 out of 31 patients (32.3%) after radiosurgery (*p* < 0.001). Detailed obliteration status in the unmatched cohort showed that for resection, 87.9% complete obliteration, 3.0% achieved near-complete (90–99%), 6.1% achieved partial (50–89%), and 3.0% had limited (< 50%) obliteration (*p* < 0.001). In contrast, the radiosurgery group showed 25.4% complete obliteration, with 22.2% achieving 90–99%, 22.2% achieving 50–89%, and 30.2% showing < 50% obliteration. Similar patterns were observed in the matched cohort, with resection showing 87.1% complete, 3.2% near-complete (90–99%), 6.5% partial (50–89%), and 3.2% limited (< 50%) obliteration, while radiosurgery resulted in 32.3% complete, 16.1% near-complete, 19.4% partial, and 32.3% limited obliteration.

**Table 2:** Comparisons of outcomes between the resection and stereotactic radiosurgery cohorts

	Original Data				Matched Data			
Characteristic	**Overall** *N* = 96^*1*^	**Resection** *N* = 33^*1*^	**Radiosurgery** *N* = 63^*1*^	**p-value** ^*2*^	**Overall** *N* = 62^*1*^	**Resection** *N* = 31^*1*^	**Radiosurgery** *N* = 31^*1*^	**p-value** ^*2*^
Complete obliteration	45 (46.9%)	29 (87.9%)	16 (25.4%)	< 0.001	37 (59.7%)	27 (87.1%)	10 (32.3%)	< 0.001
Time to complete obliteration	26.0 (12.3,45.3)	NA (NA, NA)	26.0 (12.3,45.3)		35.3 (13.8,68.9)	NA (NA, NA)	35.3 (13.8,68.9)	
Obliteration Status				< 0.001				< 0.001
Complete (100%)	45 (46.9%)	29 (87.9%)	16 (25.4%)		37 (59.7%)	27 (87.1%)	10 (32.3%)	
Near complete (90–99%)	15 (15.6%)	1 (3.0%)	14 (22.2%)		6 (9.7%)	1 (3.2%)	5 (16.1%)	
Partial (50–89%)	16 (16.7%)	2 (6.1%)	14 (22.2%)		8 (12.9%)	2 (6.5%)	6 (19.4%)	
Minimal or no obliteration (< 50%)	20 (20.8%)	1 (3.0%)	19 (30.2%)		11 (17.7%)	1 (3.2%)	10 (32.3%)	
Overall complications	35 (36.5%)	11 (33.3%)	24 (38.1%)	0.6	22 (35.5%)	10 (32.3%)	12 (38.7%)	0.6
Symptomatic Complications	34 (35.4%)	11 (33.3%)	23 (36.5%)	0.8	21 (33.9%)	10 (32.3%)	11 (35.5%)	0.8
Permanent Complications	10 (10.4%)	4 (12.1%)	6 (9.5%)	0.7	6 (9.7%)	4 (12.9%)	2 (6.5%)	0.7
Overall Rupture	7 (7.3%)	4 (12.1%)	3 (4.7%)	0.23	5 (8.06%)	3 (9.7%)	2 (6.5%)	> 0.9
Intraoperative Rupture	2 (2.1%)	2 (6.1%)	0 (0.0%)	NA	1 (1.6%)	1 (3.2%)	0 (0.0%)	NA
Post operation Rupture	5 (5.2%)	2 (6.1%)	3 (4.8%)	> 0.9	4 (6.5%)	2 (6.5%)	2 (6.5%)	> 0.9
mRS at last clinical follow up				0.4				0.3
0	34 (40.0%)	11 (40.7%)	23 (39.7%)		19 (34.5%)	10 (38.5%)	9 (31.0%)	
1	22 (25.9%)	4 (14.8%)	18 (31.0%)		15 (27.3%)	4 (15.4%)	11 (37.9%)	
2	18 (21.2%)	8 (29.6%)	10 (17.2%)		14 (25.5%)	8 (30.8%)	6 (20.7%)	
3	7 (8.2%)	3 (11.1%)	4 (6.9%)		5 (9.1%)	3 (11.5%)	2 (6.9%)	
4	2 (2.4%)	1 (3.7%)	1 (1.7%)		1 (1.8%)	1 (3.8%)	0 (0.0%)	
5	0 (0.0%)	0 (0.0%)	0 (0.0%)		0 (0.0%)	0 (0.0%)	0 (0.0%)	
6	2 (2.4%)	0 (0.0%)	2 (3.4%)		1 (1.8%)	0 (0.0%)	1 (3.4%)	
Unknown	11	6	5		7	5	2	
Last mRS vs. Presentation mRS				0.3				0.4
Better	35 (41.2%)	13 (48.1%)	22 (37.9%)		25 (45.5%)	13 (50.0%)	12 (41.4%)	
Same	41 (48.2%)	10 (37.0%)	31 (53.4%)		24 (43.6%)	9 (34.6%)	15 (51.7%)	
Worse	9 (10.6%)	4 (14.8%)	5 (8.6%)		6 (10.9%)	4 (15.4%)	2 (6.9%)	
Unknown	11	6	5		7	5	2	
Repeat treatment*	3 (3.1%)	0 (0.0%)	3 (4.8%)	0.5	0 (0.0%)	0 (0.0%)	0 (0.0%)	
Overall mortality	2 (2.1%)	0 (0.0%)	2 (3.2%)	0.5	1 (1.6%)	0 (0.0%)	1 (3.2%)	> 0.9
Mortality related to AVM	0 (0.0%)	0 (0.0%)	0 (0.0%)		0 (0.0%)	0 (0.0%)	0 (0.0%)	

^*1*^n (%); Median (25%,75%)

^*2*^Pearson’s Chi-squared test; Fisher’s exact test

*Gamma Knife radiosurgery was used for all repeat treatments

Time to complete obliteration were only available for the radiosurgery group (median 26 and 35.3 months before and after matching, respectively), as resection is generally associated with immediate obliteration or persistent residual. For the unmatched SRS cohort, the actuarial complete obliteration rates were 7.4% (95% CI: 0.1-14%) at 1 year, 20% (95% CI: 5.5-33%) at 3 years, and 51% (95% CI: 24-69%) at 5 years post-treatment (Table 3). In the matched SRS cohort, the rates were 11% (95% CI: 0-22%) at 1 year, 17% (95% CI: 0-31%) at 3 years, and 43% (95% CI: 13-63%) at 5 years post-treatment.

**Table 3:** Complete obliteration rates (95% CI) Post-SRS on radiological Follow-Up for patients with grade IV AVMs in unmatched and matched cohorts

SRS cohort	12 months	24 months	36 months	48 months	60 months
Unmatched	7.4% (0.1%, 14%)	12% (2.4%, 21%)	20% (5.5%, 33%)	33% (13%, 49%)	51% (24%, 69%)
Matched	11% (0%, 22%)	11% (0%, 22%)	17% (0%, 31%)	29% (5.3%, 46%)	43% (13%, 63%)

In univariate logistic regression analysis of matched cohorts for complete obliteration, only preoperative embolization in the radiosurgery group showed statistical significance (OR 5.83, 95% CI: 1.20–35.1, *p* = 0.036, Table 4); in the resection group it was not significantly associated with complete obliteration. No significant associations were found for age, gender, rupture status, eloquent location, nidus size, and compacted morphology in either the Resection or Radiosurgery groups.

**Table 4:** Univariate logistic regression for complete obliteration in matched cohorts

	Resection				Radiosurgery			
**Characteristic**	**N**	**OR** ^*1*^	**95% CI** ^*1*^	**p-value**	**N**	**OR** ^*1*^	**95% CI** ^*1*^	**p-value**
Age	31	1.05	0.98, 1.14	0.2	31	1.01	0.96, 1.05	0.7
Gender	31				31			
Female		—	—			—	—	
Male		0.27	0.01, 2.39	0.3		1.08	0.22, 5.06	> 0.9
Rupture	31				31			
No		—	—			—	—	
Yes		0.20	0.01, 1.77	0.2		0.89	0.18, 4.09	0.9
Eloquent location	31				31			
No		—	—			—	—	
Yes		0.00	0.00, 0.00	> 0.9		2.12	0.26, 44.8	0.5
Nidus diameter (cm)	31	1.55	0.67, 5.27	0.4	31	0.68	0.34, 1.20	0.2
Compacted	30				30			
No		—	—			—	—	
Yes		1.83	0.08, 19.3	0.6		0.25	0.03, 1.31	0.13
Preoperative embolization	31	3.50	0.36, 34.8	0.3	31	5.83	1.20, 35.1	**0.036**

^*1*^OR = Odds Ratio, CI = Confidence Interval

### Overall, symptomatic, and permanent complications

**Fig. 3:**
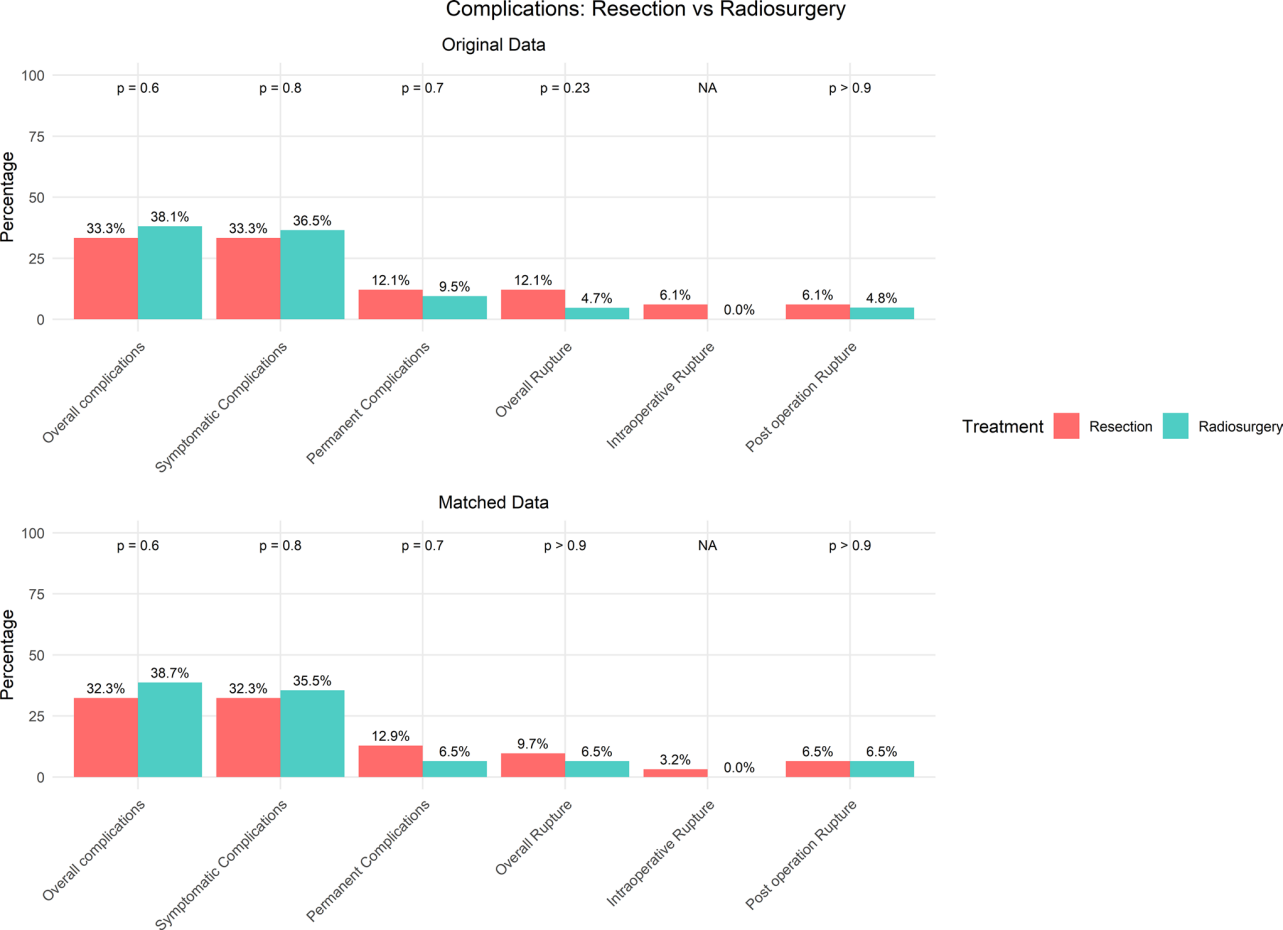
Comparison of complication between resection and stereotactic radiosurgery (SRS) cohorts, presented for both original and matched data

Both resection and radiosurgery exhibited comparable safety profiles. (Table 2; Fig. 3) Before matching, overall complication rates were 33.3% (11 out of 33 patients) for resection and 38.1% (24 out of 63 patients) for radiosurgery (*p* = 0.6). After matching, these rates remained similar at 32.3% (10 out of 31 patients) for resection and 38.7% (12 out of 31 patients) for radiosurgery (*p* = 0.6). Symptomatic complication rates showed no significant difference before matching, with 11 out of 33 patients (33.3%) experiencing complications after resection and 23 out of 63 patients (36.5%) after radiosurgery (*p* = 0.8). After matching, these rates remained comparable, with 10 out of 31 patients (32.3%) experiencing symptomatic complications after resection and 11 out of 31 patients (35.5%) after radiosurgery (*p* = 0.8). Similarly, permanent (defined as those persisting beyond 6 month) complication rates did not differ significantly between the two groups. Before matching, 4 out of 33 patients (12.1%) experienced permanent complications after resection compared to 6 out of 63 patients (9.5%) after radiosurgery (*p* = 0.7). After matching, these rates were 4 out of 31 (12.9%) for resection and 2 out of 31 (6.5%) for radiosurgery (*p* = 0.7).

In univariate logistic regression analysis of matched cohorts for symptomatic complications, only the presence of AVM-associated aneurysms in the radiosurgery group showed statistical significance (OR 7.00, 95% CI: 1.44–40.9, *p* = 0.021, Table 5). No significant associations were found for age, gender, rupture status, eloquent location, nidus size, compacted morphology, and preoperative embolization in either the Resection or Radiosurgery groups.

**Table 5:** Univariate logistic regression for symptomatic complication in matched cohorts

	Resection				Radiosurgery			
**Characteristic**	**N**	**OR** ^*1*^	**95% CI** ^*1*^	**p-value**	**N**	**OR** ^*1*^	**95% CI** ^*1*^	**p-value**
Age	31	0.99	0.94, 1.04	0.7	31	0.98	0.93, 1.02	0.4
Gender	31				31			
Female		—	—			—	—	
Male		1.10	0.24, 5.10	> 0.9		1.55	0.34, 7.11	0.6
Rupture	31				31			
No		—	—			—	—	
Yes		0.89	0.18, 4.09	0.9		2.23	0.50, 10.5	0.3
Eloquent location	31				31			
No		—	—			—	—	
Yes		2.12	0.26, 44.8	0.5		0.79	0.11, 6.87	0.8
Nidus diameter (cm)	31	1.12	0.63, 1.96	0.7	31	1.10	0.64, 1.87	0.7
Compacted	30				30			
No		—	—			—	—	
Yes		0.71	0.10, 6.17	0.7		1.43	0.31, 6.61	0.6
Aneurysm	31	0.57	0.10, 2.71	0.5	31	7.00	1.44, 40.9	**0.021**
Preoperative embolization	31	0.35	0.06, 1.91	0.2	31	2.23	0.50, 10.5	0.3

^*1*^OR = Odds Ratio, CI = Confidence Interval

### Intra- and postoperative rupture

Before matching, the overall rupture rate was 12.1% (4 of 33 patients) following resection and 4.7% (3 of 63 patients) after radiosurgery (*p* = 0.23) (Table 2; Fig. 3). In the unmatched resection cohort, 2 patients experienced intraoperative rupture (6.1%), and 2 patients had postoperative rupture (6.1%). In contrast, the radiosurgery group had 3 cases of postoperative rupture (4.8%) and no intraoperative ruptures (0%). After matching, the overall rupture rate was 9.7% (3 of 31 patients) for resection and 6.5% (2 of 31 patients) for radiosurgery (*p* > 0.9). In the matched cohort, intraoperative rupture occurred in 1 patient (3.2%) in the resection group and none in the radiosurgery group, while both groups showed equal postoperative rupture rates of 6.5% (2 out of 31 patients each, *p* > 0.9).

### Functional outcomes

**Fig. 4:**
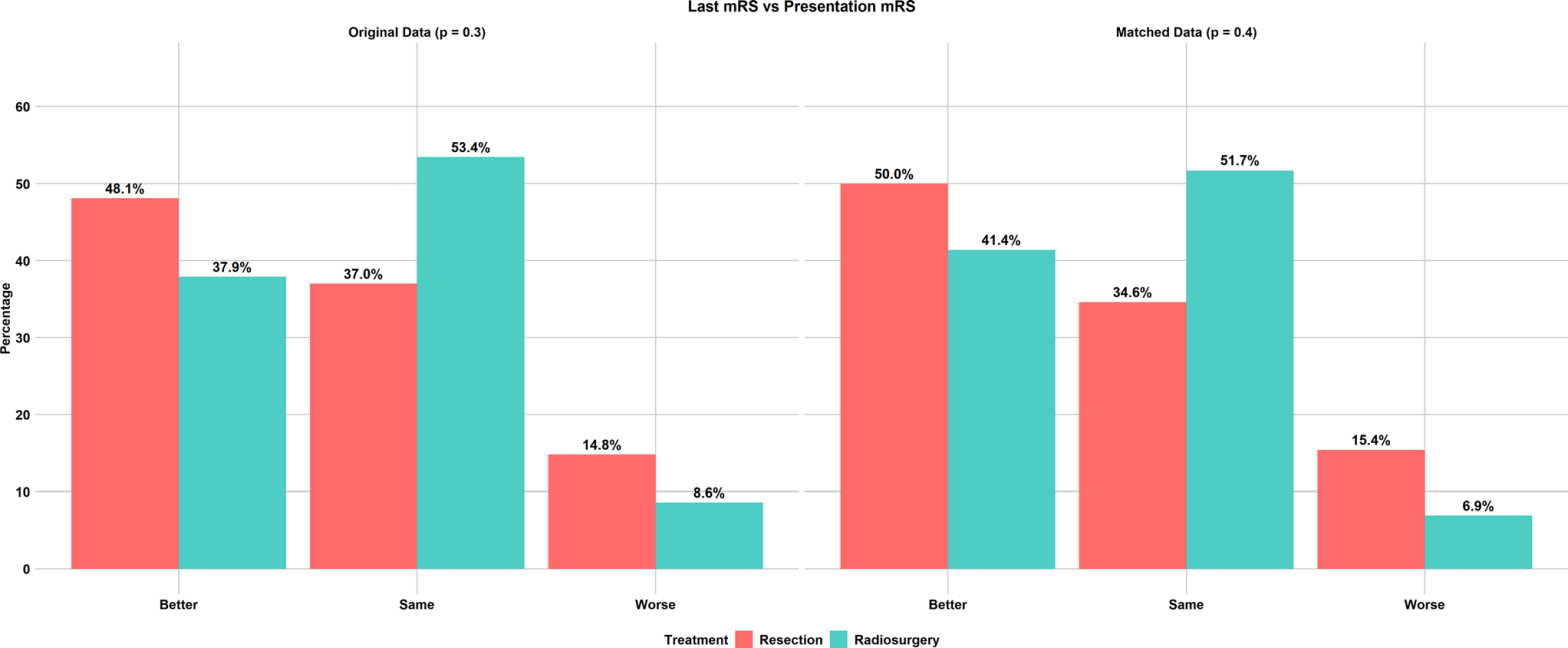
Comparison of last recorded modified Rankin Scale (mRS) scores to pre-treatment mRS between resection and stereotactic radiosurgery (SRS) cohorts, shown for original and matched data

Before matching, there was no statistically significant difference in functional outcome as measured by mRS after treatment, regardless of whether they underwent resection or radiosurgery (*p* = 0.4). (Fig. 4; Table 2) 48.1% of resection patients experienced an improvement in their mRS score, compared to 37.9% of radiosurgery patients. Meanwhile, 37.0% of resection patients and 53.4% of radiosurgery patients remained functionally the same as before treatment. Only a small percentage of patients experienced a worsening of their mRS score: 14.8% in the resection group and 8.6% in the radiosurgery group. After matching, the lack of significant difference in functional outcome changes persisted (*p* = 0.4); 50% of resection patients experienced improvement in their mRS score compared to 41.4% of radiosurgery patients, 34.6% of resection patients remained functionally stable compared to 51.7% of radiosurgery patients, and 15.4% of resection patients had worse mRS scores compared to 6.9% of radiosurgery patients. In terms of repeat treatment, 3 patients (4.8%) in the unmatched radiosurgery group required additional gamma knife radiosurgery, while no patients in the resection group needed retreatment (*p* = 0.5). After matching, no patients in either group required repeat treatment. Finally, no significant differences were observed in mortality rates related or unrelated to AVM treatment before or after matching.

## Discussion

**Fig. 5:**
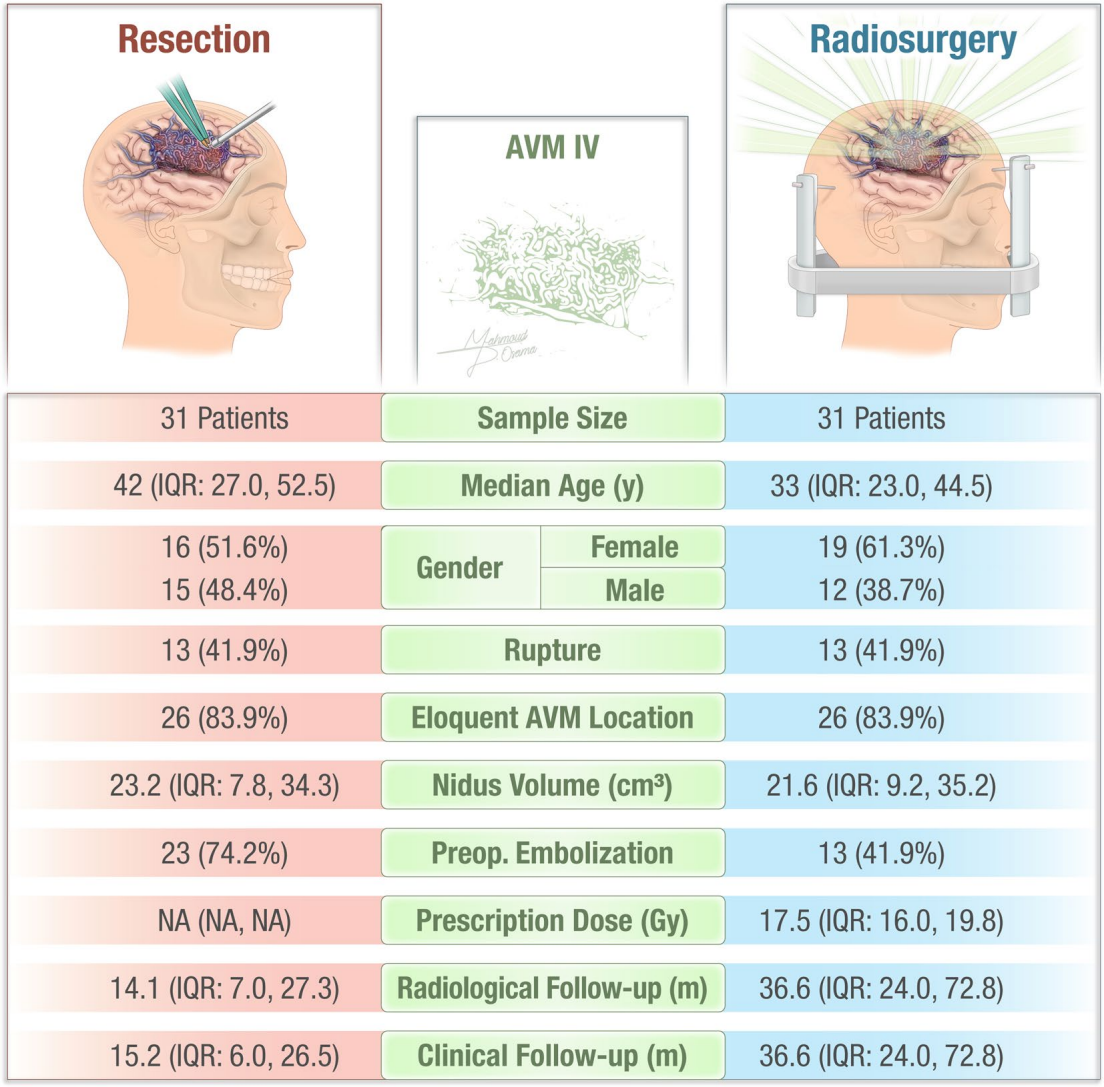
Baseline characteristics of patients undergoing resection versus stereotactic radiosurgery (SRS) for Grade IV arteriovenous malformations (AVMs)

The treatment of SM Grade IV AVMs continues to be the subject of debate in the neurosurgical community due to the inherent risks of both their natural progression and available interventions [25–27]. These lesions, characterized by their complex angioarchitecture and involvement of eloquent brain regions, pose significant therapeutic challenges. Our study aimed to address the ongoing uncertainty by directly comparing the outcomes of resection and SRS in Grade IV AVMs (Fig. 5).

### Efficacy of resection versus SRS: key findings and comparisons

Our analysis demonstrated that microsurgical resection achieved an obliteration rate of 87.1%, significantly surpassing the 32.3% achieved with SRS (*p* < 0.001). This stark contrast underscores the immediate benefits of microsurgery in obliterating AVMs, which is particularly critical in patients with a history of hemorrhage. The study by Alfter et al. echoes these findings, reporting an 82.3% obliteration rate in SM Grade III, IV, and V AVMs treated using a multimodal approach with preoperative embolization followed by microsurgical resection [12]. With regard to SRS, Ding et al. reported similar findings, showing an overall obliteration rate of 44%, with 10% at 3 years and 23% at 5 years​ [11]. The discrepancy between crude and actuarial obliteration rates in Ding et al.‘s study is noteworthy. The higher crude rate (44%) compared to the 5-year actuarial rate (23%) suggests that significant obliteration can occur beyond the conventional 5-year follow-up period typically used for treatment decision-making. This finding challenges the standard practice of recommending additional treatment at the 5-year mark for non-obliterated AVMs. It emphasizes the need for extended follow-up in SRS-treated cases and highlights the importance of individualized patient management, carefully weighing the risks of continued AVM presence against the potential for late obliteration and the risks of additional intervention. In addition, Patibandla et al. conducted an international multicenter study on SRS for Grade IV and V AVMs and reported obliteration rates ranging from 15% at 3 years to 42% at 12 years, with an overall favorable outcome (defined as AVM obliteration, no post-SRS hemorrhage, and no symptomatic radiation-induced changes) in only 26.2% of patients [13]. Further corroborating these findings, the systematic review by Graffeo et al. observed that for SM Grade IV and V AVMs, the crude obliteration rate was only 46%, again emphasizing the challenges of radiosurgery in achieving definitive outcomes in higher-grade lesions​ [14]. The variation in obliteration rates across studies may be attributed to differences in AVM sizes, radiation dosing strategies, preoperative embolization state, follow-up duration, and patient selection. The treatment of large AVMs remains a significant challenge due to their size and proximity to critical brain structures. In our study, 64.5% of patients in the radiosurgery cohort utilized single-session SRS with a median marginal dose of 17 Gy, resulting in reduced obliteration rates but acceptable complication profiles. For large AVMs (> 10 cm [3]), alternative strategies such as volume-staged radiosurgery (VS-SRS), dose-staged radiosurgery (DS-SRS), and fractionated radiosurgery have been developed to overcome these limitations [28–30]. VS-SRS involves treating sub-volumes of the AVM in multiple sessions spaced months apart, allowing for higher cumulative doses while reducing toxicity to surrounding tissues. In contrast, DS-SRS delivers lower radiation doses across multiple sessions to the entire AVM nidus, improving safety for eloquent or deep-seated AVMs. fractionated radiosurgery, on the other hand, divides the total radiation dose into smaller fractions delivered over weeks, effectively reducing radiation-induced complications [28–30]. Recent studies have demonstrated that these staged and fractionated approaches can improve obliteration rates while maintaining acceptable safety profiles, particularly for large or high-risk AVMs [28–32].

While the immediate resolution of the AVM with resection provides a considerable advantage, it is important to consider the technical demands and potential for perioperative complications, which require careful patient selection. In contrast, SRS, though minimally invasive and appealing for AVMs in eloquent or deep-seated regions, comes with the drawback of a prolonged latency period before obliteration is achieved. The median follow-up in SRS cohort was 36.6 months, consistent with prior studies reporting a latency period ranging from 36 to 144 months​ which significantly increases the risk of hemorrhage, with annual hemorrhage rates of approximately 3%.[11, 13] The risk of post-SRS hemorrhage can be as high as 9.4%, as reported by Kiran et al., especially in patients with large or deep AVMs​ [21]. The delayed nature of SRS’s therapeutic effects, coupled with this risk, presents a major limitation, particularly in hemorrhagic cases where immediate intervention may be necessary.

Preoperative embolization, frequently used as an adjunctive strategy, seeks to address some of these limitations by reducing nidus size and altering flow dynamics, thereby improving the safety and efficacy of subsequent interventions. In our analysis, preoperative embolization was significantly associated with complete obliteration in the radiosurgery group (OR 5.83, *p* = 0.036). This finding suggests that pre-treatment embolization may enhance radiosurgical outcomes by reducing AVM size and optimizing nidus targeting; the finding aligns with that reported by Blackburn et al. study which reported high obliteration rates with SRS after preoperative embolization [33]. However, this observation contrasts with the findings of Larkin et al., who reported no significant relationship between embolization and obliteration rates for VS-SRS or DS-SRS in large AVMs [34]. Their meta-analysis highlighted that embolization did not confer additional benefits in these staged approaches, emphasizing the need for individualized strategies based on AVM characteristics [34]. The discrepancy between our findings and those in the literature could reflect differences in study populations, embolization techniques, or radiosurgical protocols. For instance, the potential benefits of embolization may vary depending on whether it is used as part of a single-session or staged radiosurgical approach. While our findings support the utility of embolization in certain radiosurgical contexts, further research is warranted to clarify its role and optimize its application in both single-session and staged radiosurgical strategies.

### Complications and functional outcomes: balancing efficacy with risk

Despite the superior obliteration rates with resection, the complication rates between the two treatment modalities were comparable. In our study, 32.3% of patients undergoing resection experienced complications, compared to 38.7% in the SRS cohort. Of these complications, symptomatic complications occurred in 32.3% (10/31) of resection patients and 35.5% (11/31) of radiosurgery patients (*p* = 0.8). Permanent complications, defined as those persisting beyond 6 months, occurred in 12.9% (4/31) of resection patients compared to 6.5% (2/31) of radiosurgery patients (*p* = 0.7). While both groups demonstrated comparable rates of symptomatic complications, permanent complications were slightly higher in the resection group, potentially reflecting the immediate risks associated with open surgery. The severity and functional impact of these complications also varied; for example, neurological deficits following resection may have a greater effect on patient independence compared to transient radiation-induced effects in SRS. This is consistent with findings from a systematic review and meta‑analysis conducted by China et al., who noted that 34% of patients treated with Gamma Knife radiosurgery experienced radiation-induced changes, with permanent neurological deficits occurring in approximately only 3% of cases [15]. In addition, Patibandla et al. reported that SRS for SM Grade IV and V AVMs carried a 3% annual hemorrhage rate during the latency period, with 10.7% of patients experiencing symptomatic radiation-induced changes and 4% suffering from permanent radiation-induced changes [13]. Similarly, Ding et al. observed that about 12% of patients treated with single-session SRS experienced symptomatic radiation-induced changes, contributing to a long-term risk of post-radiosurgery clinical deterioration​ [11].

In contrast, resection, while providing immediate obliteration, presents with its own risks. For instance, Alfter et al. reported that permanent disabling neurological complications occurred in 13.2% of patients undergoing resection, with a higher risk of morbidity in older patients [12]. They also reported that 19.2% of patients experienced death or dependency (mRS ≥ 3) following microsurgical resection​ [12]. Jiao et al. further highlighted the risk of complications, documenting 35.8% short-term negative outcomes and 18.9% long-term adverse effects following microsurgical resection [35]. These comparable complication rates challenge the assumption that less invasive modalities like SRS are inherently safer; rather, both approaches carry risks that need to be weighed against their benefits.

Functional outcomes, as measured by the mRS scale, showed improvement in 50% of patients in the microsurgery group compared to 41.4% in the SRS group. However, the absolute number of patients improving was similar between the two groups (13 in the microsurgery group vs. 12 in the SRS group), and the microsurgery group had a higher number of patients with worsening mRS scores (4 vs. 2). This improvement in functional outcomes with resection is consistent with findings from Alfter et al., who demonstrated that surgery significantly reduces the risk of long-term neurological deterioration​ [12]. China et al. further reported that SRS patients were more likely to experience post-treatment hemorrhage or delayed complications such as radiation necrosis and cyst formation, which negatively impacted their functional outcomes​ [15]. The better outcomes seen in the resection may partially reflect the immediate resolution of the AVM and the reduction in future hemorrhage risk associated with microsurgical resection. However, it is important to acknowledge that the differences in outcomes could also be influenced by selection bias, as patients with AVMs located in surgically challenging areas or those with significant comorbidities were more likely treated with SRS. This selection bias, along with factors such as the severity of the initial hemorrhage, may have impacted the observed outcomes, which is a known limitation of retrospective studies like ours.

### Critical context from existing literature: the lack of comparative data

One of the critical contributions of this study is its direct comparison of resection and SRS in high-grade AVMs. Much of the existing literature focuses on single-modality studies, making cross-comparisons difficult. Studies such as those by Theofanis et al. and Kiran et al. have examined either resection or SRS in isolation [21, 36]. Our study, by employing propensity-score matching, attempts to balance comparisons between patients and provides a robust framework for understanding the relative strengths and weaknesses of both treatment modalities. A notable finding from Han et al. was the relatively low annual hemorrhage risk of 1.5% in untreated high-grade AVMs, prompting the authors to advocate for conservative management in select cases [9]. This raises an important question about the necessity of aggressive intervention in all patients, particularly in those with unruptured AVMs. However, studies by Alfter et al. and China et al. argue that multimodal treatment—especially in hemorrhagic cases—remains the most effective strategy to prevent rebleeding and achieve long-term neurological stability​ [12, 15]. Additionally, Jayaraman et al. reported an annual pretreatment hemorrhage rate of 10.4%, which dropped to 6.1% posttreatment. This reduction was significant in both patients with hemorrhagic presentation (5.6%, *P* < 0.0003) and those without (6.4%, *P* < 0.045), with the greatest reduction seen in hemorrhagic cases [2].

### Implications for clinical practice and future research

The implications of our findings for clinical practice are significant. Resection remains the treatment of choice for hemorrhagic AVMs, particularly in cases where immediate obliteration is crucial to prevent rebleeding [37–39]. The higher obliteration rates and superior functional outcomes associated with resection make it a clear option for patients who are surgically viable candidates. However, SRS plays an essential role, especially for AVMs located in deep or eloquent brain areas where the risks of resection are prohibitively high [39, 40]. The lower obliteration rates and prolonged latency period of SRS underscore the need for careful patient selection and long-term follow-up.

The successful treatment of high-grade AVMs relies heavily on both the technical expertise of the treating neurosurgeons and the institutional infrastructure. High-volume centers with a dedicated focus on AVM management are more likely to achieve favorable outcomes due to their specialized teams, well-developed protocols, and experience with complex cases. While our study focuses on treatment modalities, the potential influence of institutional expertise on outcomes should not be overlooked. The variability in center-level experience reflects real-world clinical diversity and enhances the external validity of our findings. However, future investigations should stratify results based on institutional expertise to better understand its impact on success and complication rates.

One of the key strengths of our study is the use of propensity-score matching to reduce bias and ensure comparability between the resection and SRS groups. This approach allowed us to isolate the effects of each treatment modality more accurately, providing more reliable data on obliteration rates, complications, and functional outcomes. Additionally, our study contributes valuable data on the long-term outcomes of both treatment modalities, particularly regarding time to obliteration, which is a critical factor when considering the risks of post-treatment hemorrhage.

### Limitations

Our study has several notable limitations. The most significant is the inherent selection bias in treatment decisions, particularly regarding lesion location and angioarchitecture. Despite propensity score matching, important differences persisted between the groups: the resection group predominantly included lobar lesions (particularly parietal and occipital), while the SRS group contained more deep-seated lesions (basal ganglia, thalamus, and corpus callosum) and diffuse-type AVMs (11 versus 6 cases). This reflects the clinical reality that treatment decisions in our retrospective study were influenced by lesion characteristics and safety considerations, with microsurgical resection rarely applied to deep-seated or high-grade AVMs. These inherent biases, combined with patient preferences, are a limitation of the study design and highlight the challenges of conducting randomized controlled trials in this population. A notable disparity exists in the proportion of asymptomatic cases (20% in resection group), which is unusual as resection is typically not favored for high-risk, asymptomatic AVMs. Additionally, the impact of lesion location on functional outcomes varies significantly - lesions in sensory or visual areas may have less impact on mRS scores compared to those affecting motor pathways, potentially influencing treatment selection. These differences in lesion characteristics and anatomical distribution between groups affect the interpretation of complication rates. In addition, the inclusion of large AVMs (median volume 21.6 cc) in the SRS group and their treatment predominantly in a single session (64.5% of cases) may have influenced the observed outcomes. The Spetzler-Martin grading system alone may not fully capture these nuances that influence treatment decisions and outcomes. Another potential limitation of our study is the relatively low median marginal dose of 17 Gy and the predominance of single-stage treatments (60.3%) for large AVMs with a median volume of 25 cc, which may have contributed to the lower obliteration rates observed. It is also important to recognize that a subset of patients, particularly those of advanced age, with high-grade AVMs may be managed conservatively due to prohibitively high treatment risks. Our study does not capture this population, which represents an important consideration in the overall management of high-grade AVMs. Data on pre-embolization nidus volumes were not collected and analyzed in this study, potentially limiting our ability to estimate the effect of embolization in obliteration rates. Another limitation of this study is the relatively short 13-month follow-up period for the surgery group, which may not account for long-term outcomes or late-onset complications. Future studies would benefit from more closely matched lesion locations, detailed analysis of location-specific complications, and longer follow-up periods.

## Conclusion

In this retrospective multicenter registry, resection was more effective than SRS in the obliteration of Spetzler martin grade IV AVMs, though both modalities carry significant risks. While our study suggests comparable outcomes between surgical resection and radiosurgery for high-grade AVMs, these results should be interpreted cautiously due to potential selection biases and the generalizability of study results from tertiary referral centers. A multidisciplinary approach that integrates individualized patient care is essential for optimizing outcomes, and future research should focus on refining treatment strategies and enhancing long-term follow-up.

## Data Availability

No datasets were generated or analysed during the current study.
